# Root Apex Transition Zone As Oscillatory Zone

**DOI:** 10.3389/fpls.2013.00354

**Published:** 2013-10-02

**Authors:** František Baluška, Stefano Mancuso

**Affiliations:** ^1^Institute of Cellular and Molecular Botany, Department of Plant Cell Biology, University of BonnBonn, Germany; ^2^LINV – DiSPAA, Department of Agri-Food and Environmental Science, University of FlorenceSesto Fiorentino, Italy

**Keywords:** plant roots, plant sensory biology, plant electrophysiology, plant polarity, plant morphogenesis, plant cytoskeleton, plant communication, auxin, neurotransmitter agents

## Abstract

Root apex of higher plants shows very high sensitivity to environmental stimuli. The root cap acts as the most prominent plant sensory organ; sensing diverse physical parameters such as gravity, light, humidity, oxygen, and critical inorganic nutrients. However, the motoric responses to these stimuli are accomplished in the elongation region. This spatial discrepancy was solved when we have discovered and characterized the transition zone which is interpolated between the apical meristem and the subapical elongation zone. Cells of this zone are very active in the cytoskeletal rearrangements, endocytosis and endocytic vesicle recycling, as well as in electric activities. Here we discuss the oscillatory nature of the transition zone which, together with several other features of this zone, suggest that it acts as some kind of command center. In accordance with the early proposal of Charles and Francis Darwin, cells of this root zone receive sensory information from the root cap and instruct the motoric responses of cells in the elongation zone.

## TRANSITION ZONE AS INITIATOR OF ROOT TROPISMS, CELL POLARITY, AND CELL FATE SWITCHES

Since pioneering experiments of Charles and Francis Darwin, published in their book more than 150 years ago, it is known that growing root apices are actively behaving, exploring their environment and searching for water and nutrients. Their root caps are acting as sensory organs transmitting sensory information into motoric subapical root zones (Darwin, 1880). However, as root caps are relatively far away from the elongation region, which drives the differential cell/root growth for the root tropisms, it was not obvious how the sensory root cap effectively controls root growth directions. In 1990, we revealed that a large zone, comparable in size to the meristem, is located between the apical meristem and basal elongation region (Baluška et al., 1990). Later, we reported that microtubules and F-actin filaments accomplish complex and extensive re-arrangements in cells of the transition zone (Baluška et al., 1992, 1997). In addition, it turned out that the basal border of the transition zone is very flexible and it can be regulated very sensitively by gravity. In particular, cells at the upper part of gravistimulated maize roots, especially the outer cortex cells which accomplish the basipetal (shootward) transport of auxin (Kollmeier et al., 2000), enter the rapid elongation zone sooner (closer to the root tip) as they would do if not placed into the horizontal position (Baluška et al., 1993a, b, 1996a). This results in immediate speeding-up of the root surface extension rate at the upper root part, causing roots to bend downward (along the gravity vector). This finding has twofold impacts. Firstly, it indicates that root gravitropism is not initiated, as widely believed, only by differential elongation of cells but also by differential release of transition zone cells into the region of rapid cell elongation (Baluška et al., 1993a, b, 1996a, b). Secondly, the transition zone bending starts almost immediately after the gravistimulation, suggesting that it performs sensory processes and effective sensory-motoric integration (Mancuso et al., 2006; Baluška et al., 2010a; Baluška and Volkmann, 2011). Roots exposed to exogenous auxin, which stops growth completely, are still able to initiate and develop gravitropic curvature (Ishikawa and Evans, 1993; Ottenschläger et al., 2003; Yamamoto, 2003). As the basal (rootward) border of the transition zone fluctuates between diverse root sides or cell files (Baluška et al., 1996b; Ivanov and Dubrovsky, 2013), it emerges that the transition zone cells have flexible fates. At its both the apical and basal transition zone borders, either entering it or leaving it, cells are under tight control of diverse endogenous or exogenous factors, rendering the transition zone developmental buffering capacity (Baluška et al., 2010a; Ivanov and Dubrovsky, 2013). Actually, as we are discussing it below, cellular polarities and fates can switch dramatically in this developmentally flexible root apex zone.

## TRANSITION ZONE AS INITIATOR OF ROOT HAIRS AND ORGAN MORPHOGENESIS

Epidermis and pericycle cells represent morphogenetic root tissues as they can, under proper induction, accomplish either root hair formation or lateral root primordial formation. Both root hairs and lateral roots are specified at the basal (shootward) border of the transition zone. The competence for either the root hair initiation or lateral root primordium initiation is present in within this narrow developmental window of root cell fate history. Both, developmental and ecological contexts determine how many roots hairs or lateral roots are generated in particular primary root (Osmont et al., 2007; Péret et al., 2011; Jones and Ljung, 2012). *Arabidopsis* roots, similarly as roots of other Brassicaceae species, are different with respect of root hairs because they are generated irrespective of this ecological context, strictly in the trichoblast cell files. Unique aspect of lateral root primordial formation is that new organs are initiated endogenously, deeply within the root tissues (Sablowski, 2004). All other important morphogenesis processes in plants are initiated and accomplished in the epidermis layer. However, roots of *Arabidopsis*, and other Brassicaceae, also do not enter into symbiosis with mycorrhizal fungi. Interestingly, symbiotic interfaces between fungal cells and root cells are developed in the inner cortex cells, close to the pericycle of the transition zone. Endodermis as the peripheral tissue of the stele protects against symbiotic fungi and bacteria, but not against haustorial roots of parasitic plants (Tomilov et al., 2005). Roots of some plants enjoy symbiosis with nitrogen-fixing Rhizobia bacteria when bacteria-harboring nodules resemble lateral root primordia, and initiate their development close to this root apex zone. Finally, also parasitic roots target the transition zone of host roots for their invasion (Tomilov et al., 2005).

Our study of root hair initiation in maize root epidermis revealed, that the very early step, known as bulge formation, is accomplished by local modification of cell wall, especially recruitment and activation of expansions (Baluška et al., 2000a, b). This local weakening of the cell periphery complex results in bulging out of the outer cell epidermis portion, switching the cell polarity locally, and subsequent assembly of the F-actin-based tip growth machinery, which drives the root hair formation. Similarly as with the differential root surface extension of gravistimulated roots, also root hair formation can be accomplished even when microtubules are depolymerized (Baluška et al., 1996a, 2000a). Activated tip growth machinery recruits secretory vesicles which, in addition to the Golgi-derived vesicles, rely heavily on endosomes and endocytic recycling vesicles motility of which is under F-actin control (Voigt et al., 2005a). In this respect, the root hair tip zone resembles closely the synaptic recycling domains (Baluška et al., 2005a), with the only difference being that endocytosis balances exocytosis at the root synapses, so that there is no growth or any obvious cell periphery expansion. Nevertheless, it cannot be excluded that the synaptic domains are rhythmically expanding and shrinking, as exocytosis and endocytosis processes are not tightly coordinated. Interestingly in this respect, the tip-growth domains perform inherent oscillations in root hairs and pollen tubes (Feijó et al., 2001; Monshausen et al., 2007; Rounds and Bezanilla, 2013). Furthermore, endocytic vesicle recycling is important for repair of the *stressed* plasma membranes (Baluška and Wan, 2012), as well as for the evolutionary origin of action potentials (Goldsworthy, 1982) which have a potential to protect the integrity of stressed plasma membrane (Baluška and Wan, 2012). Intriguingly, peaks of proton influxes are associated with alkalinization at the clear zone of tip-growing pollen tubes (Feijó et al., 1999), as well as with the unique status of the root apex transition zone (Collings et al., 1992; Baluška and Mancuso, 2013a). Could it be that the evolutionary origin of the transition zone is related to the stress biology of plant roots and their very symbiotic nature? Indeed, land plant evolution has been driven by symbiotic interactions of the earliest land plants with fungi and bacteria (Jorgensen, 1993; Simon et al., 1993; Wang et al., 2010; Sanders et al., 2011; Baluška and Mancuso, 2013b). Evolving plants, and especially their roots, invented very high complexity of proteins of the PIN, PILS, and ABC transporter families (Cho and Cho, 2012; Feraru et al., 2012; Viaene et al., 2013). This allowed evolution of root apex sensory-motoric circuits driving complex architecture and behavior of root systems (Baluška et al., 2009a, b; Baluška, 2012a; Burbach et al., 2012; Wan et al., 2012) exploring effectively heterogenous soil patches (Hodge, 2009; Monshausen and Gilroy, 2009; Trewavas, 2009). Especially the shootward PIN2 transporter and non-genomic auxin receptor ABP1 emerge to be essential for the evolution of plant synapses and root apex transition zone (Baluška et al., 2009a, 2010a; Baluška, 2012a; Baluška and Mancuso, 2013b).

## TRANSITION ZONE AS OSCILLATORY ZONE: FROM TRANSPORT OSCILLATIONS AT MEMBRANES, VIA DYNAMIC AND INTEGRATED CYTOSKELETON, TO OSCILLATING GENE EXPRESSION IN NUCLEUS

**FIGURE 1:**
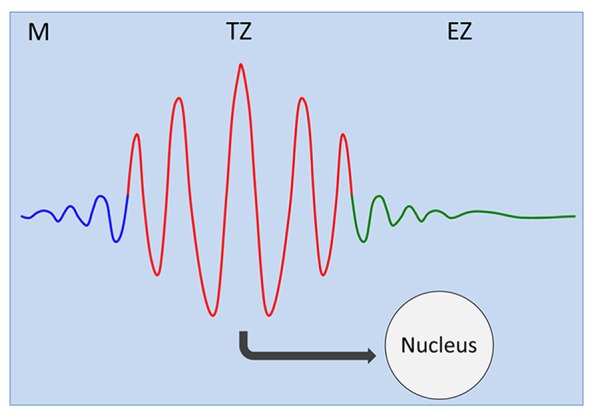
**Transition zone as oscillatory zone.** Oscillations of the plasma membrane electrical potentials generate, via multiple feedback loops with membrane associated cytoskeleton, self-sustaining and self-regulating cellular oscillator (Shabala et al., 1997, 2006; Mancuso and Boselli, 2002; Shabala, 2003; Mancuso et al., 2005, 2007; McLamore et al., 2010a, b; Mugnai et al., 2012). Oscillatory patterns of ion and auxin fluxes at the plasma membrane feed into the oscillations of gene expression in the nucleus (Moreno-Risueno et al., 2010, 2012; Traas and Vernoux, 2010; Moreno-Risueno and Benfey, 2011). M, meristem; TZ, transition zone; EZ, elongation zone.

**FIGURE 2:**
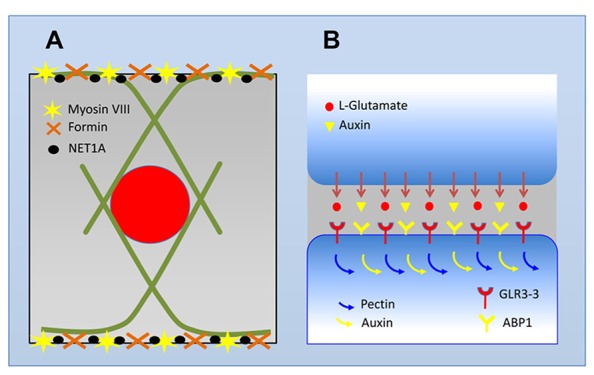
**Schematic view of the transition zone cell and its auxin-secreting synapse. (A)** Cells in the transition zone maintain their nuclei in the central position. Instead of fine F-actin networks typical for meristematic cells, these cells develop prominent bundles of F-actin which enclose the nucleus in a cage-like structures. Inhibition of endocytic vesicle recycling with brefeldin A results in a disintegration of this F-actin cage and nuclei are shifted out of their central position (Baluška and Hlavacka, 2005). At the plasma membrane, F-actin bundles are anchored at F-actin rich end-poles which are active in endocytosis/endocytic vesicle reycling. Anchoring of F-actin bundles at the plasma membrane and support of dense F-actin meshorks at these domains is accomplished via myosin VIII, group Ie formins, and NET1A actin-binding protein (Baluška et al., 1997, 2000c, 2002, 2009a; Deeks et al., 2005, 2012; Baluška, 2012a). **(B)** Abundant myosin VIII, dense meshworks of F-actin, and very active endocytosis of cell wall pectins crosslinked with boron and calcium allow tight synaptic cell–cell adhesion. At these adhesive and polar cell domains, cells secrete auxin out cells via the endocytic vesicle recycling of pectins and numerous plasma membrane proteins including putative auxin transporters of the PIN family (Baluška et al., 2002, 2008; Mancuso et al., 2005, 2007). Auxin is peceived at the adjacent cells via its plasma membrane/cell periphery receptor ABP1. Glutamate is proposed also to be secreted out of plant cells and glutamate receptor-like protein GLR3.3 is enriched at these synaptic domains (see Figure 1C in Vincill et al., 2013).

**FIGURE 3:**
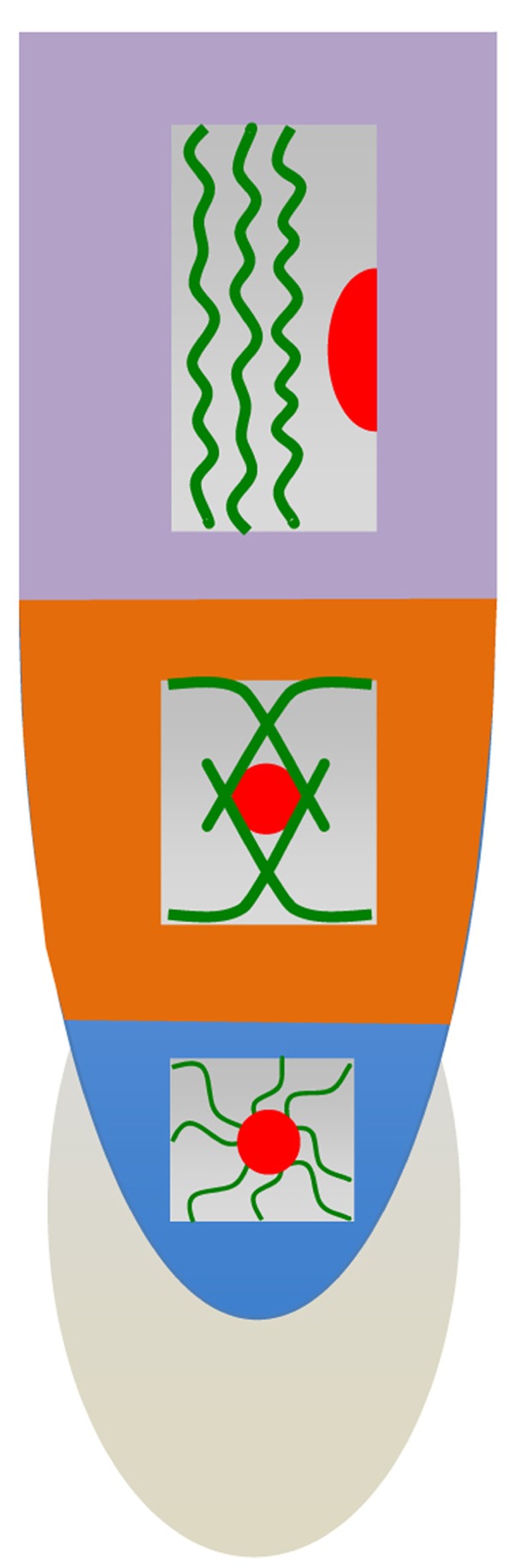
**Schematic views of cellular architecture in meristem, transition zone and elongation region.** Cells in the meristem are characterized with centrally positioned nuclei suspended in networks of F-actin and radial arrays of perinuclear microtubules (Baluška et al., 1992, 1997, 2000c; Voigt et al., 2005b). In the transition zone, nuclei still keep their central position, but fine F-actin networks are replaced by bundles of F-actin organized via the nuclear surface and the end-poles enriched with myosin VIII (Baluška et al., 1997, 2000c; Reichelt et al., 1999). In the elongation region, cells start to elongate very rapidly and develop their central vacuole which is pushing their nuclei toward the side walls. F-actin bundles obtain longitudinal and wrinkled/loosened appearances (Baluška et al., 1997, 2000c; Voigt et al., 2005b).

**FIGURE 4:**
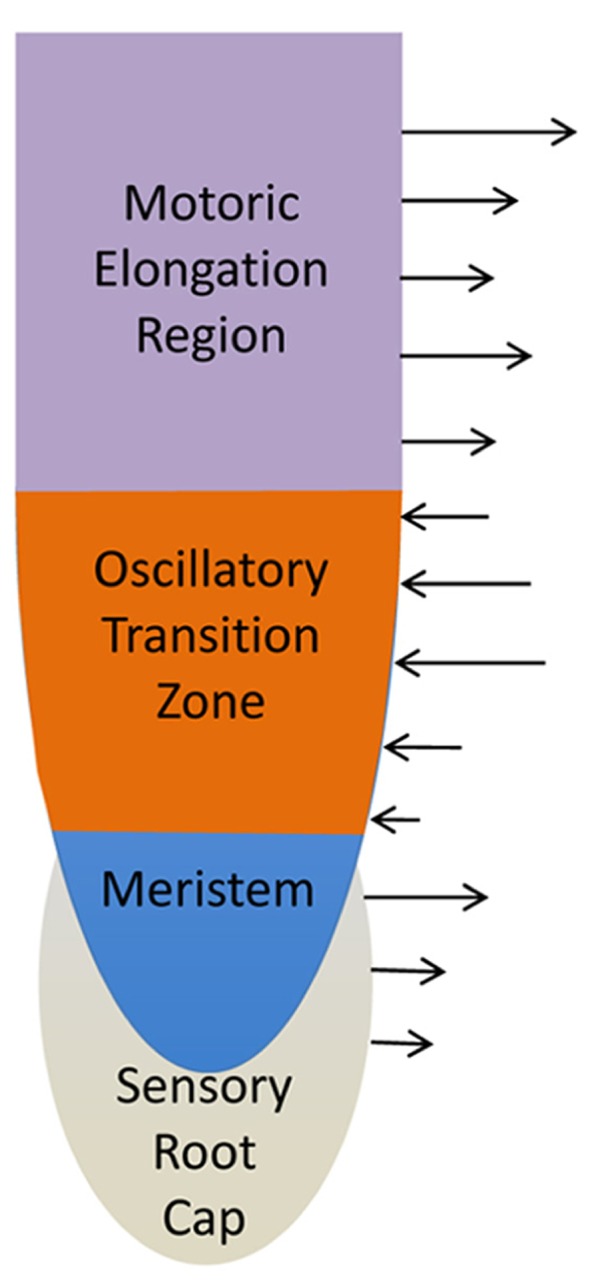
**Schematic views of cellular root apex zones and their electric fields.** Sensory root cap is enclosing the meristem with dividing cells. These two most apical zones are characterized by the outward electric current (Collings et al., 1992; Baluška and Mancuso, 2013a). Adjacent transition zone is characterized with two inversions of the electric current pattern at the root apex. The apical border of the transition zone accomplishes the outward–inward switch whereas the basal border the inward–outward switch (Collings et al., 1992; Baluška and Mancuso, 2013a). The elongation region is characterized again by the outward electric current. High root synaptic activities at the transition zone are linked with the prominent inward electric currents.

Recent addition to the unique properties of cells in the transition zone is very prominent ion transport activities. Particularly striking is the fact that ion fluxes shows not only peaks at the transition zone, but also that these show highly oscillating nature specifically in this root zone, feature which is missing in all other root zones (Shabala et al., 1997, 2006; Shabala and Knowles, 2002; Shabala, 2003; McLamore et al., 2010a, b). Moreover, besides ion fluxes, also other transport processes show peaks of their activities and oscillate specifically in the transition zone. Here we can mention oxygen influx and polar auxin fluxes (Mancuso and Boselli, 2002; Mancuso et al., 2005, 2007; McLamore et al., 2010a, b), and nitric oxide (NO) emissions (Illéš et al., 2006; Mugnai et al., 2012). As almost all plasma membrane processes investigated in the transition zone show oscillations, it can be proposed that the primary plasma membrane generated oscillations (**Figure 1**) are transferred, via dynamic cytoskeleton, up to the nucleus which is still centrally localized in cells of the transition zone (**Figures 2 and 3**). This central position of the nucleus is actively maintained via cytoskeletal connections of the nuclear surface to the plasma membrane, and is important for its information processing roles (Baluška, 1998, 2000b, 2004a, 2004b, 2006a). Synchronizations are primarily generated at the plasma membrane (**Figure 1**), and these are then perlocated, via dynamic cytoskeletal elements, up to the centrally positioned nuclei (**Figures 2 and 3**). Importantly, oscillating gene expression waves define the prebranch sites of future lateral root primordia (Moreno-Risueno et al., 2010, 2012; Traas and Vernoux, 2010; Moreno-Risueno and Benfey, 2011). In this respect, the transition zone resembles the segmentation clock of vertebrate embryos. Not only that new root primordia are formed deeply within root tissues, in a stark contrast to other examples of plant organogenesis, but they also show other striking parallels to developing animals (Traas and Vernoux, 2010). Importantly in this respect, whereas the shoot organogenesis is hard-wired and accomplished at the shoot periphery in a spiral-like patterns, the root organogenesis is soft-wired (sensitive to sensory information) and accomplished endogenously in internal tissues. Root primordia are initiated in the transition zone (De Rybel et al., 2010) as probabilistic events whose frequency is based on auxin transport (Laskowski, 2013). As we are discussing it later, the oscillating gene expression pattern in nuclei of the transition zone cells is emerging to be closely linked with the oscillations in synaptic and electric activities (g**Figures 3 and 4**) of the plasma membrane and associated recycling vesicles.

With respect of the unique endogenous origin of lateral root primordia, it is very interesting that root apex stem cells are also localized deeper within root apex tissues. Moreover, genes maintaining root stem cells specify also the root identity in the early embryo (Sablowski, 2004), and there are two asymmetric formative cell divisions which underly generation of root body from its stem cells (Scheres, 2007). Intriguingly, transcription factor SHORT-ROOT (SHR) is crucial in this respect as it moves, in association with endosomes and microtubules, from the stele cells into the endodermis and root tip of *Arabidopsis*, where it specifies the endodermal cell identity and stem cell function (Koizumi et al., 2011; Wu and Gallagher, 2012, 2013). Interestingly, root-specific genes and processes underly also regeneration in plants (Sugimoto et al., 2010). Endodermis in the transition zone has unique properties, especially with respect to polar auxin transport (PAT) via PIN3 (Marhavý et al., 2013) and gibberellin signaling (Ubeda-Tomás et al., 2008, 2009; Band et al., 2012; Shani et al., 2013), making this “inner skin-like” tissue (Alassimone et al., 2012; Baluška, 2012b) crucial with respect of the root growth control and the priming of the lateral root primordia initiation (De Smet, 2012; Marhavý et al., 2013). Besides well characterized auxins and cytokinins (Benková and Hejátko, 2009; Vanstraelen and Benková, 2012), especially crucial for the transition zone prove to be gibberellins which control cell growth processes, root apex zonation (Ubeda-Tomás et al., 2008, 2009; Band et al., 2012; Shani et al., 2013), PIN2 stability and the shootward auxin transport (Löfke et al., 2013), as well as the cellular polarity and axiality via transverse organization of cortical microtubules (Baluška et al., 1993a, b; Locascio et al., 2013). Despite the obvious differences in the shoot and root organogenesis, the same PLETHORA transcription factor clade determines patterning of both shoot and root primordia in *Arabidopsis* (Hofhuis et al., 2013). Recently, all cells of the transition zone have been reported to form symplastic domain (Benitez-Alfonso et al., 2013). This high symplastic connectivity is crucial for lateral root patterning, and might be relevant also for the synchronous oscillations characteristic for this root apex zone (**Figure 1**).

## OSCILLATING PLANT SYNAPSES: ACTIN–AUXIN OSCILLATOR MEETS AUXIN–PECTIN OSCILLATOR

All root apex cells are polarized along the apical–basal root axis which is inherently linked to the polar cell–cell transport streams of auxin (Baluška et al., 2003b, 2010a). Cortical microtubules determine tubular shape of elongation root cells and F-actin enriched at the non-growing end-poles (Baluška et al., 1997, 2001a), together with endocytic recycling networks, define the identity of apical (rootward) and basal (shootward) recycling domains (Baluška et al., 2003a, b, c, 2010a). F-actin meshworks at the end-poles (**Figure 2A**) are very abundant especially in cells of the transition zone (Baluška et al., 1997, 2009a; Baluška and Hlavacka, 2005) and their abundance correlates closely with the amounts of auxin transported across, and enriched at, these cell–cell adhesion domains (Mancuso et al., 2005, 2007; Schlicht et al., 2006). F-actin is not essential for cell expansion in the transition zone (Baluška et al., 2001b), but it is critical for both endocytosis and endocytic vesicle recycling, which is inherent part of polar cell–cell transport of auxin (Baluška et al., 2003c, 2005a, 2008; Mancuso et al., 2007). Besides abundant F-actin, also plant-specific myosin VIII (Reichelt et al., 1999) is an inherent part of this plant-specific synapses (Baluška et al., 2000c, 2001c) specialized for the sensory-mediated auxin secretion (**Figure 2B**). Plant-specific myosin VIII, enriched at plant synapses, is important for the plant endocytosis (Volkmann et al., 2003; Golomb et al., 2008; Sattarzadeh et al., 2008), being the main driver of the plant synaptic activity. Besides myosin VIII, also formins and actin-binding protein NET1A organize F-actin at the plant-specific root synapses (**Figure 2A**) of the transition zone (Baluška and Hlavacka, 2005; Deeks et al., 2005, 2012).

**FIGURE 5:**
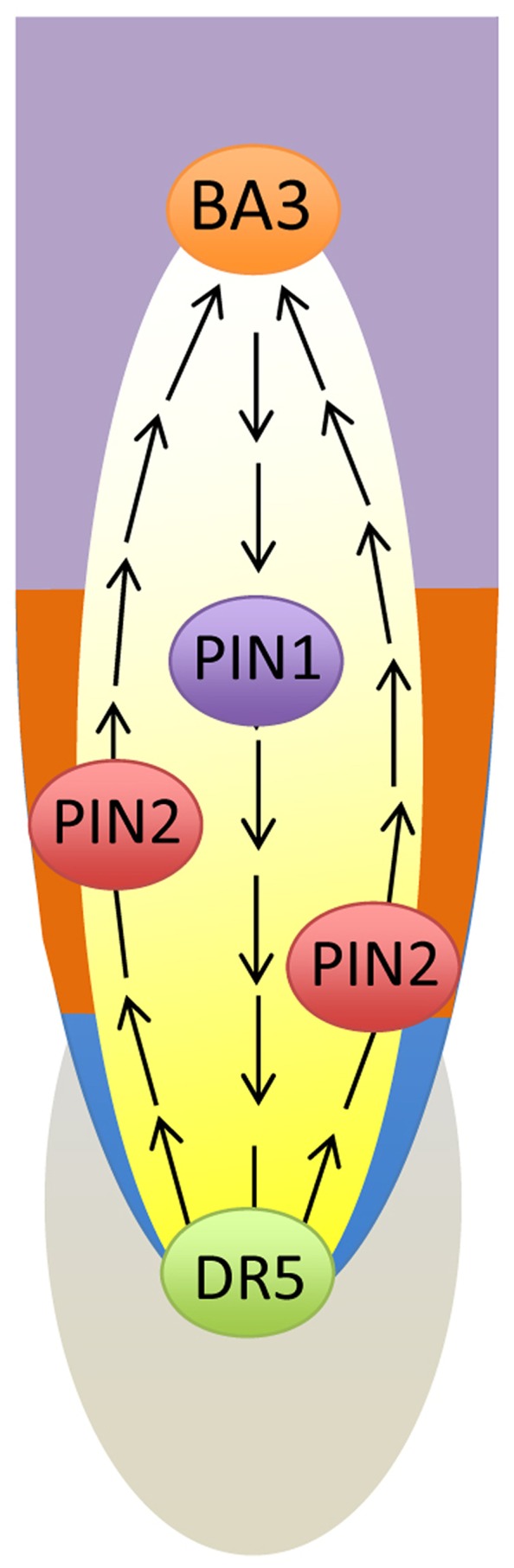
**Schematic views of two loops of the polar auxin transport streams at the root apex.** In the central stelar tissue, polar auxin transport stream is polarized toward the root cap. Here it is redistributed laterally in a fountain-like manner toward the root periphery at which the stream gets opposite (shootward) polarity and continues up to the basal border of the transition zoner, where it is looping back via an inversed fountain-like manner back to the central stelar tissues. This complex pattern of polar cell–cell transport of auxin is tightly linked with sensory events at the root cap and instruct the motoric events at the apical portion of the elongation region (Baluška et al., 2009a, b, 2010a; Baluška and Mancuso, 2013a).

Importantly, F-actin and auxin transport are connected via feedback interactions which are at the core of actin–auxin oscillations (Nick et al., 2009; Nick, 2010; Qiao et al., 2010; Durst et al., 2013). It was a big surprise when we discovered more than 10 years ago that the major cargo of endocytic recycling vesicles are cell wall pectins cross-linked with boron and calcium (Baluška et al., 2002; Yu et al., 2002). The actin–auxin oscillations characterized in tobacco BY-2 cells, organized into single cell files (Nick et al., 2009; Nick, 2010; Qiao et al., 2010; Durst et al., 2013), are apparently present also in the root apices which are composed of numerous independent but integrated cell files which are interconnected, both symplastically and electrically, via numerous cell–cell plasmodesmata (Baluška et al., 2001c, 2003a; Barlow et al., 2004; Wojtaszek et al., 2004; Šamaj et al., 2006). Similarly as the plant synapses, also these plasmodesmata, and especially their groupings known as pit-fields, are enriched with abundant F-actin/myosin VIII meshworks and active in endocytosis/endocytic vesicle recycling (Baluška et al., 2001c, 2003a, 2004c; Volkmann et al., 2003; Šamaj et al., 2006; Golomb et al., 2008; Sattarzadeh et al., 2008). Auxin is not only enriched at the active synapses (Schlicht et al., 2006), but it is also “trapped” within the recycling vesicles together with pectins and recycling PIN transporters (Šamaj et al., 2004; Schlicht et al., 2006). As inhibition of endocytosis and endocytic vesicle recycling blocks PAT in the root apex transition zone, here the auxin is acting also as plant-specific neurotransmitter (Baluška et al., 2003c, 2005a, 2008; Mancuso et al., 2007). It is secreted via synaptic-like recycling apparatus and elicits very rapid electric responses on the adjacent (postsynaptic) cells (Felle et al., 1991; Rück et al., 1993). Similarly as in neurons, endocytosis at the plant-specific synapses is controlled via the mechanical status of the plasma membrane (Baluška and Wan, 2012). Auxin represents small multifunctional signaling molecule which not only induces electric and chemical signaling cascades but also regulates endocytosis and vesicle trafficking in the post-synaptic cell (Robert et al., 2010; Baluška, 2012a; Chen et al., 2012). Obviously, auxin acts as plant-specific transmitter for effective cell–cell communication and coordination via continuous streams of the PAT conveying context-relevant sensory information (Baluška et al., 2009a, b, 2010a, b; **Figure 5**). Interestingly, similar mechano-chemical feedback regulatory loops between cell wall mechanics, controlled again via cross-linked cell wall pectins and the PAT, determine also organ formation and phyllotactic patterning in shoots (Peaucelle et al., 2011; Braybrook et al., 2012; Palin and Geitmann, 2012; Wolf et al., 2012; Braybrook and Peaucelle, 2013).

## SYNAPTIC RECYCLING OF CELL WALL PECTINS AT SYNAPTIC END-POLES: WHEN “OUTSIDE IS INSIDE”

Unique aspect of cell walls in cells of the transition zone is endocytosis and endocytic recycling of calcium/boron cross-linked pectins (Baluška et al., 2002, 2005b; Yu et al., 2002; Dhonukshe et al., 2006). Importantly, this cell wall pectin endocytosis is effective especially at microtubules depleted and F-actin and myosin VIII-enriched synaptic end-poles (Baluška et al., 1997, Baluška et al., 2001a, 2003a, b; Reichelt et al., 1999; Barlow and Baluška, 2000; Volkmann et al., 2003). These drive cell–cell transport of auxin via endocytic vesicle recycling (Baluška et al., 2003a, b, c, 2005a; Mancuso et al., 2005, 2007; Schlicht et al., 2006), resembling closely the synaptic processes of neurons in animals and humans (Baluška et al., 2003a, b, c, 2005a, 2009a, b). Interestingly in this respect, the polar cell–cell auxin transport is directly linked with sensing of environment, as well as for translating these sensory perceptions into sensory-motor circuits with the transition zone (Baluška et al., 2009a, b; Baluška, 2011; Baluška, 2012a; Baluška and Wan, 2012; Baluška and Mancuso, 2013a). In roots, both development and behavior are closely linked with sensory perceptions and sensory-motoric circuits.

It is crucial in this respect to keep in mind that the inside of endocytic vesicles corresponds topologically to the cellular outside (Baluška and Wan, 2012), so the efflux of auxin out of cells at the plasma membrane corresponds to the influx of auxin into endocytic vesicles. As auxin has one of its receptors (ABP1) exposed to the external plasma membrane leaflet (Napier, 1997; Dahlke et al., 2010), and its distribution and activities are sensitive to exogenous auxin (Diekmann et al., 1995; Tromas et al., 2009; Robert et al., 2010; Sauer and Kleine-Vehn, 2011; Shi and Yang, 2011; Xu et al., 2011; Baluška, 2012a; Chen et al., 2012; Lin et al., 2012; Wang et al., 2013), the vesicular secretion of auxin in cells of the transition zone fits well to the neurotransmitter-like concept of auxin (Baluška, 2012a). In strong support of the neurotransmitter and synaptic views of auxin in plants (Baluška et al., 2003c, 2008; Mancuso et al., 2005, 2007), auxin exerts electric responses when added exogenously to plant cells (Felle et al., 1991; Rück et al., 1993) and these electric responses are ABP1-dependent (Rück et al., 1993). Recent advances in our understanding of ABP1 suggest that this ancient protein (Tromas et al., 2009) regulates, besides the electric responses of plant cells to auxin, endocytosis, and endocytic vesicle recycling (in other words, synaptic activities), also Rho GTPase and receptor-like kinases (RLKs) signaling cascades at the plasma membrane of plant cells (Tromas et al., 2009; Robert et al., 2010; Sauer and Kleine-Vehn, 2011; Shi and Yang, 2011; Xu et al., 2011; Baluška, 2012a; Chen et al., 2012; Lin et al., 2012; Wang et al., 2013). This synaptic view of endocytic recycling and auxin cell–cell transport is relevant for both sensory and motor actions of the root apex. In this sensory-motor integration, electric activities emerge to play a central role (Masi et al., 2009).

## TRANSITION ZONE AS CENTER OF SUPRACELLULAR SYNAPTIC AND BIOELECTRIC OSCILLATING ACTIVITIES. RELEVANCES FOR THE DARWIN’S “ROOT-BRAIN” HYPOTHESIS

Our recent discoveries revealed that the transition zone, although negligible with respect of cell growth, is the most active zone in the whole root apex with respect of oscillating electric spike activities (Masi et al., 2009), endocytosis-driven vesicle recycling (Mancuso et al., 2005, 2007; Schlicht et al., 2006), and oxygen demands (Mancuso et al., 2000; Mugnai et al., 2012). Electric activity peaks at the transition zone (Collings et al., 1992; Masi et al., 2009; Baluška and Mancuso, 2013a; **Figure 4**), and perhaps this makes this root apex zone also for an attractive target of pathogenic and symbiotic organisms (Miller et al., 1986). Until now, these synaptic and electric activities represent a mystery, but these would be rather expected on the basis of the Darwin “root-brain” hypothesis (Baluška et al., 2009b; Kutschera and Niklas, 2009; Sahi et al., 2012) first postulated by Charles and Francis Darwin more than 150 year ago (Darwin, 1880; Barlow, 2006). Francis and Charles Darwin, despite having rather simple “country-house” experimental conditions (Kutschera and Briggs, 2009) accomplished relevant experiments which clearly documented that root apex behaves as *brain-like* organ, resembling brains of lower animals (Darwin, 1880; Barlow, 2006; Baluška et al., 2009b; Kutschera and Niklas, 2009). But the leading botanists of that time, Julius Sachs (Baluška et al., 2009b; Kutschera and Niklas, 2009) and Wiesner (1881) argued that experiments performed by Francis and Charles Darwin were flawed. Emil Detlefsen, assistant of Julius Sachs, as well as Julius Wiesner with the assistance from Hans Molisch claimed to demolish the fault results of Francis and Charles Darwin that the root apex acts as sensory organ controlling root tropisms accomplished in remote growth regions (Wiesner, 1881; Detlefsen, 1882). But it turned out that Francis and Charles Darwin were correct and that, in fact, the experiments of Wiesner, Molisch, Detlefsen, and Sachs were flawed, and obviously performed just to demolish the sensory plant science of Francis and Charles Darwin. Interestingly, Francis Darwin succeeded in turning-down this criticism in 1882, few days after his father death (Darwin, 1882). In fact, later studies documented clearly that the decapped maize roots grow even faster as the intact roots (Juniper et al., 1966; Pilet, 1971, 1972); also because these roots stop to crawl but grow straightly (Baluška et al., 2009a; Burbach et al., 2012). Unfortunately, this important response paper by Francis Darwin from 1882 went almost unnoticed by the botanical mainstream. If it would be taken into account, it might be that the modern plant sciences would develop in rather different directions, including sensory plant biology and root tip-based sensory control of plant root movements.

## FROM ALZHEIMER, VIA PARKINSON TO SCHIZOPHRENIA: LESSONS FROM THE TRANSITION ZONE?

The toxicity of aluminum in both plant and animal cell biology is well established, although poorly understood. Aluminum toxicity is one of the most important limiting factor for crop production in acid soils worldwide (Panda et al., 2009). Aluminum is highly toxic to root apices, with the transition zone representing the target of aluminum toxicity (Sivaguru and Horst, 1998; Sivaguru et al., 1999). Surprisingly, aluminum is less toxic to root cells which entered the elongation regions (Sivaguru and Horst, 1998). Importantly, aluminum inhibits basipetal auxin transport (Kollmeier et al., 2000), as well as PIN2 endocytosis (Illéš et al., 2006; Shen et al., 2008), endocytic vesicle recycling of PIN2 (Shen et al., 2008; Amenós et al., 2009), as well as polarity and cell patterning in the root apex (Doncheva et al., 2005), and root behavior (Poschenrieder et al., 2009).

We have reported that aluminum is internalized into the most sensitive cells of the distal portion of the transition zone in *Arabidopsis* root apices (Illéš et al., 2006; see also Babourina and Rengel, 2009), while aluminum also inhibits endocytosis in these cells (Illéš et al., 2006; Shen et al., 2008; Amenós et al., 2009). Intriguingly in this respect, elongating root cells are not sensitive to aluminum (Sivaguru and Horst, 1998; Sivaguru et al., 1999) and there is no internalization of aluminum into elongating root cells (Illéš et al., 2006; Babourina and Rengel, 2009). In support of the endocytosis/endocytic vesicle recycling being the primary processes affected in root cells, endocytosis of aluminum and its toxicity is lowered in the *Arabidopsis* mutant over-expressing of a DnaJ domain protein auxillin which regulates the clathrin-based endocytosis (Ezaki et al., 2006). Moreover, aluminum toxicity affected also NO production which is highest in cells of the distal portion of the transition zone (Illéš et al., 2006; Mugnai et al., 2012). Plant synapses being very active in endocytosis and transporting auxin show highest activities in the transition zone and it might turn out that the active plant synapses represent the aluminum target in the root apices (Panda et al., 2009; Poschenrieder et al., 2009; Baluška, 2010). This scenario is strongly supported by our finding that aluminum causes strongest depolarization of the plasma membrane potential exactly in cells of the root apex transition zone (Illéš et al., 2006). This effect is known to be mediated by glutamate and glutamate receptors (Sivaguru et al., 2003). Importantly in this respect, similarly as auxin, also L-glutamate is amino acids derived transmitter molecule which is released from synapse in form of well-defined quanta (defined by the size of synaptic vesicles). This quantal nature of cell–cell communication (Edwards, 2007) seems to be central for the very high effectivity of synapses in the cell–cell communication. This principle could also represent the basis of the still elusive flux sensor of the PAT (Merks et al., 2007; Stoma et al., 2008; Baluška, 2012a; Walker et al., 2013) which is essential for the canalization concept of PAT of Tsvi Sachs (Sachs, 1969; Sablowski, 2013).

Interestingly, in animals and humans, neuronal cells are extremely sensitive toward aluminum which is also internalized specifically in these cells (Guy et al., 1990). Aluminum was found to be enriched in lysosomes, similarly like the Alzheimer’s amyloid β-peptide plaque depositions (Schuurmans Stekhoven et al., 1990). These are also internalized from cell surface and aluminum was reported to inhibit their degradation (Sakamoto et al., 2006). Therefore, in both root cells and brain neurons, endocytosis of aluminum emerges to be relevant to its high cytotoxicity (Illéš et al., 2006; Babourina and Rengel, 2009; Kawahara and Kato-Negishi, 2011). Recent advances in plant cell biology revealed similarities between root cells sensitive to aluminum and neurons (Baluška, 2010). Further studies on these cells might give us crucial clues not just for the plant sciences but also for our understanding of the Alzheimer disease (Kawahara, 2005; Kawahara and Kato-Negishi, 2011). Plants might turn out to be useful also for our better understanding of the Parkinson disease, of the most common neurodegenerative diseases. Protein DJ-1 which is important for the onset of Parkinson disease (Lev et al., 2006), but its role is still not well understood (Aleyasin et al., 2007; Kahle et al., 2009), is present also in plants and, similarly as in neurons, regulates the reactive oxygen species (ROS) homeostasis (Xu and Møller, 2010; Xu et al., 2010). Besides plant-specific roles in chloroplast development (Lin et al., 2011), DJ-1 is expressed also in *Arabidopsis* root apex cells and its expression increased when roots are exposed to light and initiate ROS burst and light-escape tropism (Yokawa et al., 2011, 2013; Burbach et al., 2012). Finally, our understanding of schizophrenia might profit from *Arabidopsis* because the BLOS-1 protein, homologous to human and mice BLOC-1 complex BLOS proteins which are relevant for the schizophrenia (Mullin et al., 2011), controls endocytic vesicular recycling of PIN2 and PIN1 in the transition zone of *Arabidopsis* roots, and modulates their growth (Cui et al., 2010).

## TRANSITION ZONE AS EXPENSIVE, PRIVILEGED, AND PROTECTED ROOT APEX ZONE

Brains are the most expensive tissue of animals and humans (Mink et al., 1981; Navarrete et al., 2011; Howarth et al., 2012). For instance in humans, brain represent only about 2% of human body but consumes about 20% of all oxygen demand (Mink et al., 1981). Very high energy budged, due to energetically costly processes such ion channels activities, endocytosis and endocytic vesicle recycling, and dynamic actin cytoskeleton, results in very high demands for oxygen and ATP (Attwell and Laughlin, 2001; Laughlin, 2001; Harris et al., 2012). Updated energy budgets for the neural computation in mammalian neocortex and cerebellum neurons report 50% of signaling energy is used on glutamate receptors, 21% on action potentials, 20% on plasma membrane resting potentials, 5% on neurotransmitter secretory release, and 4% on vesicle recycling (Howarth et al., 2012). Surprisingly, often overlooked is also the very high ATP demand for F-actin dynamics and rearrangements (Bernstein and Bamburg, 2003).

**FIGURE 6:**
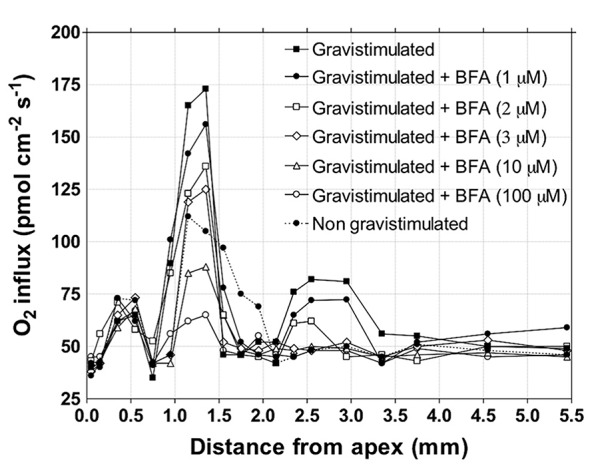
**BFA inhibits oxygen influx peak into the transition zone.** Effect of different concentrations of BFA on the profiles of the oxygen influx for the upper side in maize roots growing vertically for 5 min after gravistimulation (elicited by rotating the chamber 90^°^ until the root was horizontal. Values are means, *n* = 15. Oxygen fluxes were measured with a vibrating oxygen-selective microelectrode following the method described in Mancuso et al. (2000).

Similarly in plants, the highest demand for oxygen has been scored in the root apex transition zone, although these cells do not divide or grow significantly (Mancuso and Boselli, 2002; McLamore et al., 2010a; Mugnai et al., 2012). Moreover, importantly, inhibition of the endocytic vesicle recycling using brefeldin A lowers oxygen demand of the transition zone of maize root apices dramatically (**Figure 6**). This finding suggests that the major sink of the extra-need of these cells is tightly linked to high rates of endocytosis and of endocytic vesicle recycling at plant synapses of the transition zone (Baluška et al., 2003c, 2005a; Mancuso et al., 2005, 2007). Currently favored view is that the transcellular PAT is driven by the plasma membrane-based efflux and influx transported of the PINs and AUXs protein families. However, as a matter of fact, cells of transition zone are secreting auxin via the endocytic vesicle recycling (Mancuso et al., 2005, 2007). Inhibition of endocytic vesicle recycling via brefeldin A resembles effects of classical PAT inhibitors such as NPA and TIBA. Moreover, the PAT inhibition is accomplished sooner as removal of PINs from the plasma membrane (Baluška et al., 2003c, 2005a, 2008; Mancuso et al., 2007). Moreover, also genetic evidences support the conclusion that the PAT peak in the transition zone is based on vesicular synaptic recycling (Mancuso et al., 2007). BFA-mediated inhibition of synaptic-like vesicle recycling lowers high oxygen demand in this zone (**Figure 5**). In other words, in cells of the transition zone, the PAT does not depend on the mere presence of PINs at the plasma membrane, but rather on the high rate of endocytic recycling of vesicles loaded with auxin, its transporters, cell wall pectins, and other recycling molecules (Baluška et al., 2003c, 2005a, 2008; Šamaj et al., 2004; Mancuso et al., 2007). All this fits well with the synaptic concept of PAT (Baluška et al., 2003a, 2005a; 2008; Mancuso et al., 2007). The most active plant synapses evolved in the transition zone, which is strategically well-placed at the sites of phloem unloading. The cells of the transition zone are flooded with extracellular sucrose (Baluška et al., 2001c) inducing the fluid-phase endocytosis (Baluška et al., 2001c, 2006c; Etxeberria et al., 2005; Baroja-Fernandez et al., 2006). In cells of the maize root transition zone close to the unloading phloem, sucrose is internalized via the F-actin–myosin VIII driven fluid-phase endocytosis is active here (Baluška et al., 2001c, 2004c). Intriguingly in this respect, action potentials control both phloem long-distance transport and unloading of sucrose (Fromm, 1991; Fromm and Bauer, 1994; Fromm and Lautner, 2006); and plant glutamate receptors (GLRs)-like proteins GLR3.2, GLR3.3, and GLR3.4 are strongly expressed in *Arabidopsis* root phloem, especially near their unloading sites at the transition zone (Vincill et al., 2013). It emerges that the phloem long-distance transport and phloem unloading of sucrose are controlled electrically, perhaps via electric signals emerging from the brain-like transition zone (Masi et al., 2009). All 20 GLRs of *Arabidopsis* are expressed in their roots (Chiu et al., 2002; Roy et al., 2008) and it can be expected that they control plant-specific synaptic plasticity in the root apex transition zone, similarly as in animal brains (Lau and Zukin, 2007; Paoletti et al., 2013). Recently, GLR3.3 has been reported to be critical for the plant defense and immunity signaling (Li et al., 2013; Manzoor et al., 2013; Mousavi et al., 2013). Importantly, the GLR3.3 is not only localized at root synapses of the transition zone (see Figure 1C in Vincill et al., 2013), but it is also essential for electrical signaling that rapidly induce defense responses at remote leaves after local herbivore attack (Christmann and Grill, 2013; Mousavi et al., 2013).

Similarly like brain, also the transition zone is not only privileged but also highly protected niche. Under diverse stress situations, stress-induced abscisic acid (ABA) and NO protect cells of the transition zone. NO protects transition zone against aluminum or cadmium toxicity (Wang and Yang, 2005; Tian et al., 2007; Xiong et al., 2009; He et al., 2012) and oxygen deficit (Mugnai et al., 2012), whereas ABA protects the transition zone against water stress (Saab et al., 1990; Sharp et al., 1994; Ober and Sharp, 2003).

## AUXIN AS PLANT-SPECIFIC TRANSMITTER: SIMILARITIES TO L-GLUTAMATE AND OTHER AMINO ACIDS-DERIVED NEUROTRANSMITTERS AND NEUROMODULATORS

Auxin is chemically very close to others monoamine transmitters, especially to serotonin and melatonin. Moreover, although auxin is a very small molecule below the size limit of the plasmodesmata transport, it is not transported across these plant-specific cell–cell channels. Obviously, there are selective processes active around plasmodesmata orifices, which prevent auxin to pass freely these cytoplasmic channels between plant cells (Baluška et al., 2003c, 2006a, 2008; Wojtaszek et al., 2004). All this suggests that auxin acts as plant-specific transmitter in plants. Interestingly, monoamine transmitters control adaptive behavior in lower animals such as nematodes (Donnelly et al., 2013), resembling behavior of plant roots. In lower and higher animals, monoamine transmitters and neuromodulators orchestrate chemical codes for behavioral patterns (Bicker and Menzel, 1993; Roeder, 2005; Pirri et al., 2009; Harris-Warrick, 2011; Donnelly et al., 2013). Ability of plants to rapidly modify their behavior in accordance with the environmental challenges is essential for their survival. This principle is very obvious in plant roots, as these are movable despite of the sessile nature of higher plants. Interestingly in this respect, stressed roots accomplish effective escape tropisms if faced with unfavorable environment (Yokawa et al., 2011, 2013; Burbach et al., 2012; Wan et al., 2012). This resembles the escape behavior of nematodes (Pirri et al., 2009; Donnelly et al., 2013). Auxin is chemically very similar to monoamine transmitters such as serotonin, melatonin, dopamine, tyramine, noradrenaline, and other; which control behavior and emotions in animals and humans (Pirri et al., 2009; Lövheim, 2012; Donnelly et al., 2013). Plants synthesize all these monoamines. Although the role of these is still not understood, at least serotonin and melatonin have physiological and perhaps also neuronal-like roles in plants (Ramakrishna et al., 2011; Pelagio-Flores et al., 2011, 2012; Park and Back, 2012). Similarly as auxin, both serotonin and melatonin regulate root system architecture (Pelagio-Flores et al., 2011, 2012; Park and Back, 2012). This resembles also the effects of another transmitter L-glutamate which acts also via neuronal GLRs of plants. It can be expected that the complex behavior of plant roots is controlled not only by auxin but also by other monoamine transmitters and neuromodulators. Recent paper reports end-pole/synapse localization of the GLR3.3 in roots of *Arabidopsis*, whereas GLR3.2 and GLR3.4 are enriched especially in root apex phloem elements, especially and at their sieve plates (Vincill et al., 2013). Interestingly, all these GLRs are also involved in the control of lateral root primordia initiation as revealed by increased amounts of primordia, which are aberrantly placed (Vincill et al., 2013). In addition, the GLR3.3 not only localize to the auxin secreting synapses of the transition zone (Baluška et al., 2005a, 2008; Mancuso et al., 2007), but is also important for root gravitropism (Miller et al., 2010). Rice GLR3.1, which is belonging to the same GLR clade, was found to be essential for root growth via control of the transition zone of rice root apex, which was highly reduced in mutant roots (Li et al., 2006). Finally, L-glutamate and GLRs control also root system architecture via impacts not only on the lateral roots but also via control of the primary root growth (Walch-Liu et al., 2006; Forde and Walch-Liu, 2009). One possibility is that L-glutamate and GLRs control root growth and root system architecture via phloem transport and unloading. As already mentioned, GLRs are enriched in phloem, especially at the sieve plates (Vincill et al., 2013), close to the root apex where phloem is unloaded (Baluška et al., 2001c). Importantly in this respect, both the phloem transport and the phloem unloading are controlled electrically via action potentials (Fromm, 1991; Fromm and Bauer, 1994; Fromm and Lautner, 2006).

## PERSPECTIVES AND OUTLOOK

In contrast to the shoot apex, the plant root apex is highly regularly organized with clearly defined zones (**Figures 2 and 3**) and regular cell files (Ishikawa and Evans, 1995; Baluška et al., 1996b, 2006b, 2010a; Verbelen et al., 2006; Ivanov and Dubrovsky, 2013). It is very interesting that this anatomical zonation is associated with clear patterns of electric activities when root cap and elongation region show outward electric currents (**Figure 4**), whereas meristem and especially the transition zone generates prominent inward electric currents (Collings et al., 1992; **Figure 4**). Importantly, these electric activities not only peak but also oscillate in the transition zone (Masi et al., 2009), where also auxin fluxes oscillate (McLamore et al., 2010a, b), and the root growth rates are tightly linked with the electric currents oscillations (Souda et al., 1990; Hecks et al., 1992). Anoxia or ether exposures block the currents oscillations (Yoshida et al., 1988) and root growth (Hecks et al., 1992), whereas recovery of the root growth is linked with the re-appearance of electric currents oscillations (Hecks et al., 1992). Furthermore, oscillatory patterns of ion transport activities and electric currents at the transition zone of barley root apices are closely linked with the rhythmic patterns of nutrient acquisition (Shabala and Knowles, 2002). In future studies, it will be important to find out further details of this oscillatory strategy for root nutrient acquisition. On the other hand, these root apex-specific electric currents can feed back not only into growth processes but also into morphogenesis of root apex as cell division patterns in maize root apices are sensitive to the extracellular electric fields (Wawrecki and Zagórska-Marek, 2007). In plants, these studies are very few and our knowledge extremely limited. But in animals, it is getting obvious that the emerging bioelectric code is at least as important for the development, embryogenesis and regeneration as the genetic code (Tseng and Levin, 2013). Any living cells, and even their organelles, generate their own electric fields which feedback into the biochemical and molecular processes underlying the cell/tissue polarities (McCaig et al., 2009; Yao et al., 2009; Zhao et al., 2012; Tseng and Levin, 2013). Endocytosis emerges to be sensitive to electric fields. Similarly as in neurons, endosomes of plant cells also process the sensory information for motoric outcomes (behavior) of their organs such as roots (Baluška and Volkmann, 2011; Baluška and Wan, 2012; Wan et al., 2012).

Electric fields generated by the oscillatory transition zone resemble encephalograms scored around brains of animals and humans. These prominent electric fields represent summation of synchronous electric activities of transition zone cells, and suggest two important features of this unique root apex zone. Firstly, the transition zone cells have the highest electric activities from all cells of the whole root (Masi et al., 2009) and; secondly, this activity is highly synchronous (**Figure 1**). Only this synchronicity allows vibrating probes to detect prominent electric currents which form the characteristic pattern of the smaller outward current peak at the meristematic zone and the prominent inward current peak at the transition zone (Baluška and Mancuso, 2013a). It is still mystery why the polarity of electric current suddenly switches from outward to inward at the meristem–transition zone border (**Figure 4**). Interestingly, the inward current switches again, back to the outward current at the transition zone–elongation region border (Collings et al., 1992; Baluška and Mancuso, 2013a). This pattern of electric field was scored in roots of all species tested so far, suggesting that there is something special about the inward current of the transition zone (Baluška and Mancuso, 2013a). As it correlates with high activities of root synapses, it might be possible that sensory stimuli/experiences will modify patterns of root electric fields. In support of this attractive possibility, root apex electric fields change during gravistimulation of maize roots (Collings et al., 1992). In addition, the PAT (**Figure 5**) appears to act as both the sensor and instructor for the adaptive root behavior (Baluška et al., 2009a, b, 2010a, b). The transition zone electric field is affected by inhibitors of PAT (Collings et al., 1992). In future, it will be important to analyze the possible connections between the synaptic activities and the patterns of the electric fields around the oscillatory transition zone (**Figures 1 and 4**). Particularly, how do the sensory inputs modify these electric field patterns, and if modified electric field patterns are related to the motoric output of driving the root behavior. Sensory perceptions and active behaviors are linked to neurons and neuronal systems in both lower and higher animals. Currently, the mainstream of plant sciences considers plants for passive automata-like organisms, lacking any sensory-motoric circuits. But this position, which is based on ancient Aristotelian worldviews, depriving plants of cognition-relevant sensitivities and activities are not tenable anymore (Brenner et al., 2006, 2007;Trewavas, 2007, Trewavas, 2009; Barlow, 2008; Baluška, 2009; Calvo and Keijzer, 2010; Karpiński, 2010; and Szechyńska-Hebda, 2010;; Trewavas and Baluška, 2011; Marder, 2012, 2013; Debono, 2013). Especially root behavior shows cognitive features which are not possible to explain with currently dominated views of passive, automata-like organisms (Baluška et al., 2010b; Barlow, 2010a, b; Gagliano et al., 2012).

In humans and animal brains, neuronal oscillations are important for processing of sensory information (Schroeder and Lakatos, 2008; Uhlhaas et al., 2009, 2010; Hipp et al., 2011; Arnal and Giraud, 2012). Synchrony and oscillatory patterning of anatomically grouped neurons drive sensorimotor networks in animals. Similarly in the root apex transition zone, all cells show synchronous oscillations (**Figure 1**) which are related to root behavior based on plant-specific sensorimotor networks. In future, it will be important to understand possible links between the oscillations at the plasma membrane and gene expression oscillations, as well as the relevance of these oscillations for the *brain-like* command center status of the transition zone and the whole root apex (Darwin, 1880; Barlow, 2006; Baluška et al., 2009b). It is emerging that besides the plant-specific molecules such as auxin, also many of the classical neuronal molecules including glutamate, GABA, serotonin, melatonin, acetylcholine, will be relevant in this respect.

## Conflict of Interest Statement

The authors declare that the research was conducted in the absence of any commercial or financial relationships that could be construed as a potential conflict of interest.
